# Ledipasvir/Sofosbuvir Eradicates Hepatitis C in an Immunodeficient STAT3-GOF Patient

**DOI:** 10.1007/s10875-021-01011-9

**Published:** 2021-03-29

**Authors:** Julian Thalhammer, Maria Elena Maccari, Oliver Wegehaupt, Stephan Ehl, Carsten Speckmann

**Affiliations:** 1 Faculty of Medicine, Center for Pediatrics and Adolescent Medicine, Medical Center, University of Freiburg, Mathildenstr 1, 79106 Freiburg, Germany; 2 Faculty of Medicine, Center for Chronic Immunodeficiency (CCI), Medical Center - University of Freiburg, Institute for Immunodeficiency, University of Freiburg, Freiburg, Germany

To the Editor:

We report on the successful eradication of hepatitis C virus (HCV) by direct-acting antiviral therapy (DAA) with Ledipasvir/Sofosbuvir in an immunodeficient 10-year-old girl with a pathogenic and symptomatic signal transducer and activator of transcription 3 gain of function (STAT3-GOF)-mutation.

The girl was born at term as the second child of non-consanguineous parents in Albania and had recurrent episodes of autoimmune-hemolytic anemia (AIHA) and immune thrombocytopenia (ITP) since the age of 18 months. The initial AIHA/ITP episodes were treated primarily with repeated blood transfusions in her country of origin. Documentation of further initial clinical details including results of Coombs test or auto-antibody screenings remain unknown. At the age of 3 years, she experienced a prolonged episode of AIHA, for which she received multiple transfusions, but also required transient systemic immunosuppressive therapy (IST) with oral steroids. Results of a bone marrow puncture supported the diagnosis of AIHA/ITP. At this time, infectious disease screening identified for the first time an active HCV infection, without quantitative assessment. In the absence of other risk factors, and with her family having tested negative, the infection was most likely related to the previous blood transfusions, a common medical problem in Albania before centralized PCR testing was introduced in 2016 [1].

At the age of 5 years, the family moved to Germany, and, at the age of 7 years, the girl presented to our center with another episode of ITP that had not responded to an intravenous immunoglobulin (IVIG) application (1 g/kg), in the local primary hospital. She displayed mild facial dysmorphic features, i.e., enlarged rotated ears, thin lip vermilion, bilateral epicanthus, squinting, and discrete brachydactyly. Furthermore, mild developmental delay of motor function and speech was recognized. Size (46th percentile) and weight (61st percentile) were normal for her age. Mild splenomegaly was noted on ultrasound, but the patient showed no peripheral lymphadenopathy. Peripheral blood and bone marrow were well compatible with ITP. Molecular cytogenetic evaluation of the bone marrow was without pathological findings. The girl had a positive direct Coombs test, but no active AIHA at this time. There was no history of susceptibility to infections. Immunological studies showed normal IgA and IgM levels. IgG levels and vaccination responses were not informative due to recent IVIG treatment. Immunophenotyping showed normal total lymphocyte and CD3+ T cell count. Yet naïve CD45RA+ T-cells and naïve B-cells were reduced. Activated CD4+ and CD8+ T-cells and CD21 low B-cells were expanded and NK-cell numbers were slightly reduced. For full details, see Table S1. Quantitative PCR revealed an HCV load of 4.7 × 10^6^ IU/ml (Abbott®). Laboratory and sonographic liver studies showed normal liver function and anatomy.

After repeated, yet again ineffective, IVIG administrations (3 × 1 g/kg), oral steroid therapy (prednisone 2 mg/kg/d) induced remission of ITP (Fig. 1). An inborn error of immunity (IEI) was assumed in the context of chronic bilinear autoimmune cytopenia, splenomegaly, and dysmorphic features. She was enrolled in our center’s AL-PID study and a genetic diagnosis of germline STAT3-GOF (c.373C>G; p.Gln125Glu) was established by whole exome sequencing and analysis on a panel of 230 known IEI genes. A 17-year-old brother is clinically healthy. After genetic counseling, the family decided against mutation analysis of further family members. The patient’s missense mutation leads to increased transcription of STAT3 but does not change phosphorylation (data not shown) and was observed in another patient of our cohort and functionally analyzed within our AL-PID study [2].

**Fig. 1:**
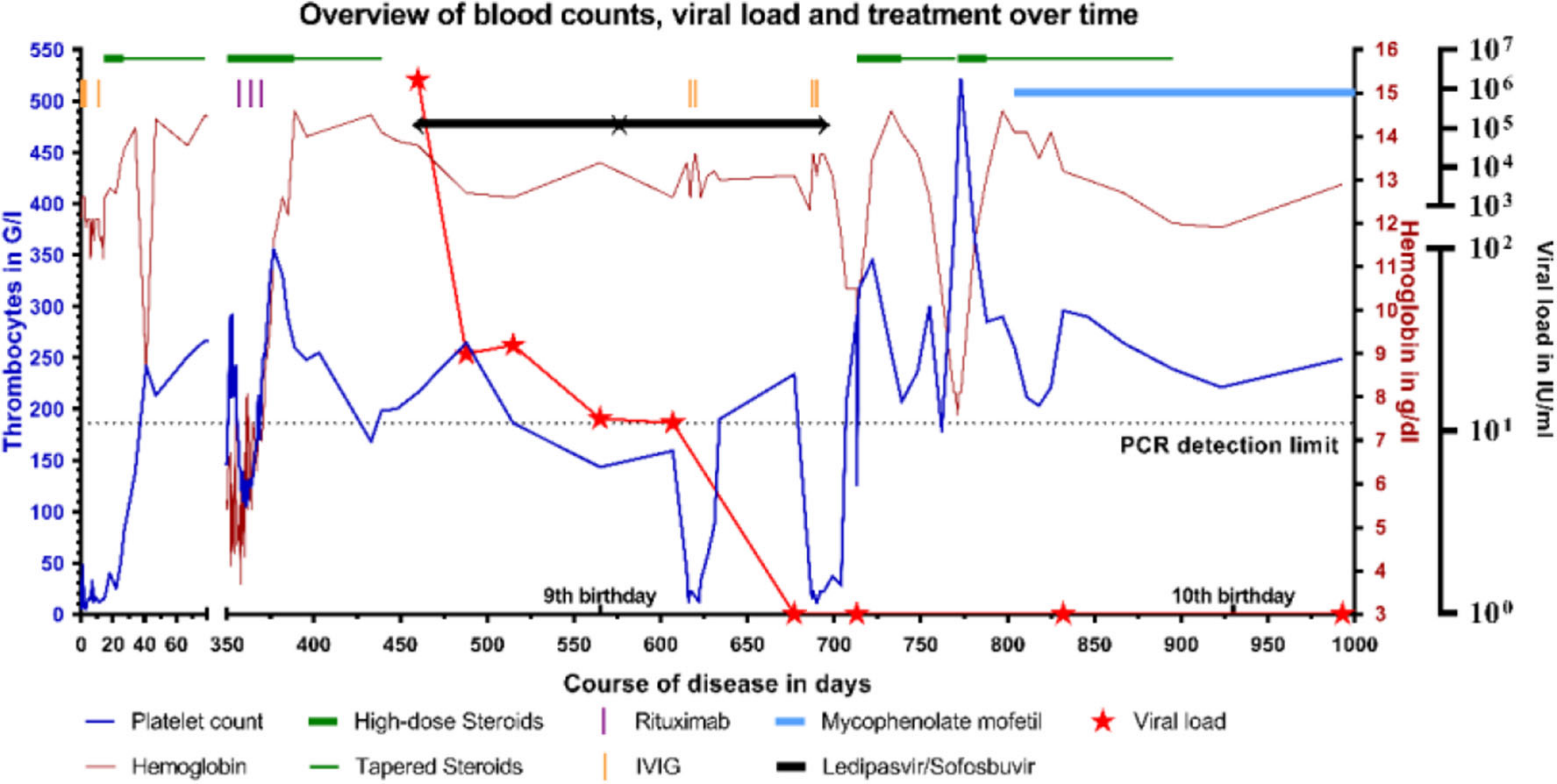
Overview of blood counts, viral load, and treatment over time. The platelet count in cells/μl (blue, left *Y*-axis) and the hemoglobin count in g/dl (red, middle *Y*-axis) are shown over time after initial presentation at our hospital. The *X*-axis is interrupted from day 80 to day 350, when the course was uneventful. Various treatments of episodes of ITP and AIHA are depicted. Thick green line shows 2 mg/kg/day prednisone treatment and thin green line shows prednisone tapering. Orange line signifies 1 g/kg IVIG, purple line 375 mg/m^2^ rituximab, and light blue 1200 mg/m^2^/day mycophenolate mofetil. The black, right *Y*-axis shows the viral load in IU/ml assessed by quantitative PCR (Abbott®), which is depicted with red stars and a red line. DAA with Ledipasvir/Sofosbuvir 45/200 mg once daily is shown as black line. Detection limit of PCR is 12 IU/ml and after borderline positivity on day 565 and day 607 was permanently negative thereafter. DAA was continued for 24 weeks corresponding to a treatment total of 168 days

STAT3-GOF (OMIM #15952) is an IEI activating signal transduction and thus leading to often early-onset multi-organ autoimmunity. The phenotype and penetrance are highly variable. Eighty-three percent of patients have hematologic involvement, mostly in the form of autoimmune cytopenia and often in combination (57% ITP, 45% AIHA, 21% autoimmune neutropenia) [2]. STAT3-GOF causes pathological lymphoproliferation in around two-thirds of patients. Susceptibility to mostly respiratory infections is present in half of the patients. Hypogammaglobulinemia (42%) and changes in T-, B-, and NK-cell number and differentiation are common [3].

Six months after the genetic diagnosis at the age of 8 years, the patient had a first life-threatening episode of AIHA with a minimal Hb of 3.4 g/dl despite transfusion of 15 packed red blood cells (PRBCS) over 3 weeks. Initial therapy with oral prednisone 2 mg/kg/day was not successful, yet 3 doses of weekly rituximab (375 mg/m^2^/week) led to remission (Fig. 1).

The combination of aggravating autoimmune cytopenia, requiring repeated IST in a STAT3-GOF patient with potentially further decreasing T-cellular immunity over time, raised our concerns about the long-term control of the patient’s chronically active HCV infection. In addition, chronically active virus diseases are contraindications for certain 2nd and 3rd line treatment strategies in severely symptomatic STAT3-GOF patients, i.e., broader immunosuppression by JAK-inhibitors (Jakinibs) or hematopoietic stem cell transplantation (HSCT). Genotyping of HCV revealed genotype 1B in our patient, for which treatment options changed dramatically over the last years. Ledipasvir/Sofosbuvir induces sustained viral responses and received initial approval for adults in the USA in 2014 [4]. Ledipasvir/Sofosbuvir is a combination of a nucleotide analogue inhibitor of the HCV NS5B polymerase and a HCV NS5A inhibitor, which directly act against the HCV. In 2018, Murray et al. reported well-tolerated and effective direct antiviral therapy with Ledipasvir/Sofosbuvir in otherwise healthy children >6 years of age [5]. In February 2019, our patient started an oral DAA off-label treatment with 45/200 mg Ledipasvir/Sofosbuvir once daily at a viral load of 10^6^ IU/ml. After 28 days of DAA therapy, viral load dropped to 30 IU/ml and remained borderline positive after 12 weeks, which is the standard treatment interval for Ledipasvir/Sofosbuvir (Fig. 1). Due to residual viral replication, we extended DAA therapy for another 12 weeks. No clinical or laboratory side effects were observed, yet the general clinical course continued to be complicated by flares of ITP, which we attributed to the underlying disease. ITP was successfully treated with repeated IVIG applications to avoid additional IST. After 21 weeks of antiviral treatment, HCV PCR turned negative for the first time. After completion of a 24-week cycle of Ledipasvir/Sofosbuvir, oral steroids had to be re-administered for recurrent ITP. Immunomodulatory therapy was changed to oral mycophenolate mofetil (1200 mg/m^2^/day) during steroid taper. One year later, no further cytopenia occurred and the patient remains negative for replicating HCV, notably despite ongoing IST after the end of DAA. Recently, in June 2020, Ledipasvir/Sofosbuvir was also approved by the European Medicine Agency for use in otherwise healthy children older than 3 years.

DAA achieves sustained viral responses in almost all adult patients with acquired immunodeficiencies such as human immunodeficiency virus (HIV) or IST after kidney transplantation. Historic cases show a high disease burden and mortality of HCV infection in patients with IEI and data on treatment with DAA of those patients, especially children, are scarce [6]. Overall, we see no major concerns regarding the general safety of Ledipasivir/Sofosbuvir, but like to point out with our report, that successful treatment is even possible in immunocompromised pediatric patients—although prolonged therapy cycles might be necessary. Even challenged by continuous IST, HCV eradication by DAA proved to be permanent in our patient. We therefore encourage the earliest possible evaluation for DAA in HCV-infected children and adults with inborn errors of immunity. DAA might be less effective with progressive exacerbation of the underlying primary or treatment-associated secondary immunodeficiency, resulting in a significant risk for loss of viral control and subsequent development of liver cirrhosis and hepatocellular carcinoma.

## Supplementary Information

ESM 1 (DOCX 25 kb)
