# Supplementary material for: Ledipasvir/Sofosbuvir Eradicates Hepatitis C in an Immunodeficient STAT3-GOF Patient
Source: J Clin Immunol. 2021 Mar 29;41(6):1365–7. doi: 10.1007/s10875-021-01011-9 (PMC8310846; doi:10.1007/s10875-021-01011-9)
Supplement: Supplementary file 1 — (DOCX 25 kb) [file 10875_2021_1011_MOESM1_ESM.docx]

Supplement

Supplementary table S1: Immunological studies at diagnosis.

| **Test [unit]** | **Number** | **Reference range** |
| --- | --- | --- |
| Leukocytes [/µl] | 4400 |  |
| Lymphocytes [/µl] | 2201 | 1100 – 5900 |
| Lymphocytes [%] | 50.00 | 35.00 – 45.00 |
| CD3+ T-cells [/µl] | 1664 | 700 – 4200 |
| CD3+ T-cells [%] | 75.60 | 55.00 – 78.00 |
| CD3+CD4+ T-cells [/µl] | 969 | 300 – 2000 |
| CD3+CD4+ T-cells [%] | 44.00 | 27.00 – 53.00 |
| CD3+CD8+ T-cells [/µl] | 646 | 300 – 1800 |
| CD3+CD8+ T-cells [%] | 29.40 | 19.00 – 34.00 |
| CD4/CD8-ratio | 1.50 | 0.90 – 2.60 |
| CD56+CD16+ NK-cells [/µl] | 61 | 90 – 900 |
| CD56+CD16+ NK-cells [%] | 2.80 | 4.00 – 26.00 |
| DR+ in CD3+ T-cells [%] | 25.40 | 5.00 – 9.50 |
| DR+ in CD3+CD4+ T-cells [%] | 21.20 | 3.00 – 13.00 |
| CD45RA+ in CD3+CD4+ T-cells [%] | 10.40 | 46.00 – 77.00 |
| CD45RO+ in CD3+CD4+ T-cells [%] | 87.50 | 13.00 – 30.00 |
| DR+ in CD3+CD8+ T-cells [%] | 32.10 | 6.00 – 29.00 |
| CD19+ B-cells [/µl] | 421 | 200 – 1600 |
| CD19+ B-cells [%] | 19.10 | 10.00 – 31.00 |
| IgM++CD38++ transitional B-cells [%] | 4.67 | 2.00-30.00 |
| IgD+CD27- naïve B-cells [%] | 39.00 | 62.00-94.00 |
| IgD+CD27+ of B-cells [%] | 18.72 | 4.00-24.00 |
| IgD-CD27+ of B-cells [%] | 8.77 | 3.00-18.00 |
| IgA+CD27+ memory B-cells [%] | 2.27 |  |
| IgA+CD27- atypical IgA+ memory B-cells [%] | 0.26 |  |
| IgG+CD27+ memory B-cells [%] | 0.87 |  |
| IgG+CD27- atypical IgG+ memory B-cells [%] | 0.39 |  |
| CD21-CD38- of B-cells [%] | 26.98 | 1.00 – 17.00 |
| IgD-IgM+CD27+ of B-cells [%] | 8.18 | 0.50 – 8.00 |

Supplementary legend

Supplementary Table 1

Flow cytometric analysis of lymphocytes. High values are red and low values are blue. (Reference range (10th and 90th percentiles) according to Schatorje: Scand J Immunol. 2011, Hulstaert: Clin. Immunol. 1994., and Shearer: JACI 2003)
